# Cardiac Rehabilitation in Patients with Peripheral Artery Disease—A Literature Review in COVID-19 Era

**DOI:** 10.3390/jcm11020416

**Published:** 2022-01-14

**Authors:** Razvan Anghel, Cristina Andreea Adam, Dragos Traian Marius Marcu, Ovidiu Mitu, Florin Mitu

**Affiliations:** 1 Clinical Rehabilitation Hospital, Cardiovascular Rehabilitation Clinic, Pantelimon Halipa Street nr 14, 700661 Iasi, Romania; razvan0312@gmail.com (R.A.); adam.cristina93@gmail.com (C.A.A.); mitu.florin@yahoo.com (F.M.); 2 Department of Internal Medicine, University of Medicine and Pharmacy “Grigore T. Popa”, University Street nr 16, 700115 Iasi, Romania; dragos.marcu11@yahoo.com; 3 “Sf. Spiridon” Clinical Emergency Hospital, Independence Boulevard nr 1, 700111 Iasi, Romania

**Keywords:** cardiac rehabilitation, peripheral artery disease, COVID-19, intermittent claudication, quality of life, review

## Abstract

Cardiac rehabilitation (CR) is an integral part of the management of various cardiovascular disease such as coronary artery disease (CAD), peripheral artery disease (PAD), or chronic heart failure (CHF), with proven morbidity and mortality benefits. This article aims to review and summarize the scientific literature related to cardiac rehabilitation programs for patients with PAD and how they were adapted during the COVID-19 pandemic. The implementation of CR programs has been problematic since the COVID-19 pandemic due to social distancing and work-related restrictions. One of the main challenges for physicians and health systems alike has been the management of PAD patients. COVID-19 predisposes to coagulation disorders that can lead to severe thrombotic events. Home-based walking exercises are more accessible and easier to accept than supervised exercise programs. Cycling or other forms of exercise are more entertaining or challenging alternatives to exercise therapy. Besides treadmill exercises, upper- and lower-extremity ergometry also has great functional benefits, especially regarding walking endurance. Supervised exercise therapy has a positive impact on both functional capacity and also on the quality of life of such patients. The most effective manner to acquire this seems to be by combining revascularization therapy and supervised exercise. Rehabilitation programs proved to be a mandatory part of the integrative approach in these cases, increasing quality of life, and decreasing stress levels, depression, and anxiety.

## 1. Introduction

**Figure 1:**
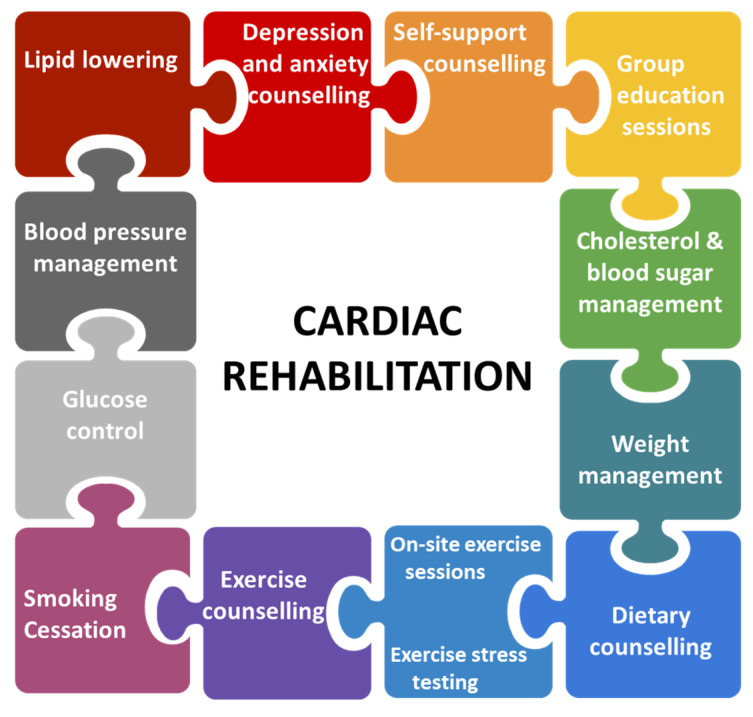
Components of cardiac rehabilitation.

Cardiovascular diseases (CVD) are still the main cause of mortality and morbidity despite a downwards trend due to earlier diagnostic facilities and advanced interventional techniques. Cardiac rehabilitation (CR) (alongside risk-factor management) is an integral part of the management of various CVD such as coronary artery disease (CAD), peripheral artery disease (PAD), chronic heart failure (CHF) or valvular heart disease (VHD), with proven morbidity and mortality benefits [1]. Physical exercise is an important element of rehabilitation, but in order to obtain a secondary prevention through CR we need to focus also on other aspects such as dietary advice, psychotherapy, smoking cessation programs, weight management or drug therapy. Improving functional capacity, psychological adaptation to chronic illness, applying lifestyle changes or maintaining the independence of daily activities are central objectives in CR to favorably influence the long-term prognosis (Figure 1) [2,3,4]. This article aims to review and summarize the scientific literature related to current cardiac rehabilitation programs for patients with PAD and how they were adapted during the COVID-19 pandemic through telemedicine. Besides that, in the light of the information available to date, we have assessed the effectiveness of home-based CR programs for patients with PAD who face multiple challenges in the context of the COVID-19 pandemic. Different databases were exploited and brief descriptions of the different types of exercise are presented in this article. From pathophysiology to comparisons between traditional and novel exercise types, benefits of CR programs and challenges due to the COVID-19 pandemic, all of these issues are discussed in terms of efficacy and prognostic value.

## 2. Literature Research

We conducted a search using the PubMed and SCIENCE DIRECT in November 2021 using the terms and phrases, “Coronavirus”, “COVID-19”, “cardiac rehabilitation”, “peripheral artery disease” and “exercise training” under different word associations. We focused on studies related to the impact of COVID-19 on cardiovascular rehabilitation programs. For inclusion, we selected observational cohort studies of patients with PAD previously confirmed by an ABI less than 0.9 or using imaging methods (duplex ultrasound, angiography) or with a history of lower-limb endovascular or open-surgical revascularization. Interventional studies, case reports, editorials and letters were excluded.

## 3. Cardiac Rehabilitation during the COVID-19 Pandemic

The implementation of CR programs has been problematic since the COVID-19 pandemic due to social distancing and work-related restrictions (“smart working”). Worldwide CR programs have been interrupted or have been reduced as they are considered non-essential in the pandemic emergency [5].

The suspension of CR based centers has significant consequences for both shorter- and longer-term prognoses as well as increased rates of acute coronary events and hence exposure to infection [6]. The appearance of mental health problems, increasing sedentary lifestyle, inadequate nutrition and giving up or decreasing regular physical activities are factors associated with negative long-term consequences [5,7]. The occurrence of PAD before the age of 50 years is currently defined as premature PAD and unfortunately remains undertreated despite the continued increase in prevalence [8,9]. Both the medical and economic impact of COVID-19 infection increases with age and the number of associated comorbidities. Wu and McGoogan pointed out that the mortality rate increased from 8.0% in 70–79 years old patients to 14.8% in patients over 80 years old [10]. Given the prevalence of CVD in patients over 65 years old, with great impact on both morbidity and mortality, the improvement in health status secondary to CR programs is essential.

Virtual CR (VCR) is an alternative for center-based CR programs, with the same patient outcomes and safety profiles for cardiac patients with low to moderate cardiovascular risk. Tele-medicine alternatives-telephone, video conferencing and other resources are some of the methods used in VCR [11].

There are some disadvantages such as resource limitations, communication difficulties, as well as risk stratification and supervision associated issues especially in elderly and frail cardiovascular patients with several associated comorbidities. Risk stratification is important in both traditional CR programs and VCR, especially when patients associates residual ischemia, reduced left ventricular ejection fraction (less than 40%), arrhythmias, or variable hemodynamic responses induced by exercise [12,13].

Italy has been one of the most affected countries by the COVID-19 pandemic. There, the Italian Association of Preventive and Rehabilitative Clinical Cardiology (AICPR) conducted a national survey on the activity of CR centers during the COVID-19 pandemic. They collected information from 75 centers (one-third of the national network) and showed that 14% of CR units continued their activities without any reorganization, 61% reduced or reshaped their programs and 25% completely stopped all of their activities. Half of CR units have implemented programs dedicated to COVID-19 patients and were managed by a multidisciplinary team that involved various health specialties (especially physiatrists and pulmonologists) [14].

Besides Italy, Canada is another country which has experienced negative impacts of the COVID pandemic on their health system. The Canadian Cardiac Rehabilitation Association implemented special home-based CR programs for PAD patients with low and moderate risk. With limited hospital visits and counselling via telemedicine, the patients completed their exercise programs in a safe and familiar environment [15,16,17,18]. At the beginning of the COVID-19 pandemic, 41.2% of the CR centers closed due to restrictions and staff redeployment and therefore a large proportion of PAD patients discontinued their CR programs. The inclusion criteria have also been modified, with patients with less complex pathologies, less motor disorders and without severe cognitive disorders being enrolled [19].

The need for alternative delivery models such as tele-cardiology, home CR programs or the use of digital health tools that allow for increased access and participation has never been more urgent. Patients with pre-existing cardiovascular diseases who have contracted COVID-19 must be considered a priority for CR centers, especially due to the long-term impact of the virus on cardiovascular and pulmonary function. As the CR programs have been reduced or interrupted worldwide due to the pandemic, we should very carefully balance the decision of excluding the high-risk patients from VCR, as studies have shown benefit of appropriately prescribed physical activity in these patients [20]. Patient education and independence are key elements of self-manage for PAD patients. The role of telerehabilitation has become increasingly important in the context of COVID-19 through the rapid development of online education programs designed to replace traditional exercise programs.

Anderson et al. demonstrated in a Cochrane Database Systematic Review that home-based exercise programs and center groups CR programs have the same results in terms of mortality, occurrence of acute cardiac events, improvement of physical parameters and correction of risk factors, with greater adherence for the first category [20]. The concept of telehealth improved during the COVID-19 pandemic and nowadays has become a relevant alternative to traditional CR programs. Through telephone, internet or videoconferencing, telerehabilitation is a cost-effective method, impacting the quality of life and mortality rate of PAD patients [21,22,23]. Telerehabilitation has both advantages and limitations. Greater independence, lower costs and privacy are factors that increase patient adherence to CR programs, while the lack of social contact, the need for at least a minimal knowledge of technology in the digital era and data security and confidentiality can be perceived as barriers [24,25,26].

The target of the VCR programs should be reaching the expected functional benefit with the least level of physical activity and with exercise intensity lower than moderate. Two important aspects of VCR are the patient’s education regarding symptom and intensity assessment which can be achieved through heart rate check-up by palpation or by using wearable heart rate monitors as well as communication with the specialists from the CR center who coordinates the exercise [17,20].

O’Doherty et al., in association with the British Association for Cardiovascular Prevention and Rehabilitation, conducted an international study based on a mixed methods survey, which aim was to investigate whether CR centers for PAD patients continued their exercise programs during the COVID-19 pandemic. Approximately half of the centers noted that CR programs were stopped due to COVID-19. The telephone was most used in VCR, as well as teleconferencing in centers where both physicians and patients had access to this type of advanced technology. The study also identified some limitations regarding the impact of technology in exercise-based CR programs such as lack of patient confidence, technical difficulties (patients without access to computers/tablets/smartphones or internet connection), concerns about patient safety (from both professionals and patients) or internet security and patient confidentiality concerns [17,27,28,29,30]. Both physical and psychological benefits of exercise training depend on the weekly exercise time and long-term adherence to the program [31].

Patients with PAD infected with COVID-19 require rigorous supervision due to the persistent systemic inflammatory state, defined as, “long COVID” [32]. Cardiac rehabilitation through physical exercises adapted to each patient and carefully supervised are essential after COVID-19 in order to decrease endothelial inflammation [33]. The 6 Minute Walking Test (6MWT) is easy and simple to use during recovery from COVID-19 in order to evaluate the physical function and to identify any potential deficiencies in daily life activities. During exercise, saturation, blood pressure, heart rate and worsening of symptoms need to be permanently evaluate (Table 1) [34,35,36,37,38].

COVID-19 has multiple implications on the morbidity and mortality of patients with PAD. SARS-CoV2 infection causes the appearance of a pro-inflammatory status accompanied by cytokine storms that cause secondary vascular inflammation and endothelial dysfunction. SARS-CoV2 infection can cause arterial and venous thromboembolic complications in patients with previous CVD [39,40,41]. Patients with PAD and COVID-19 may develop acute limb ischemia secondary to thrombosis of the aorta or peripheral arteries secondary to endothelial dysfunction [41,42]. Both PAD and COVID-19 causes hypercoagulability, but it is currently not well defined whether PAD patients associate worse outcomes when infected with SARS-CoV2. Smolderen et al. demonstrated that PAD patients have an increased risk of 40% when it comes major averse cardiovascular events in case of co-existence of infection with COVID-19 [43].

Enrolling these patients in cardiac rehabilitation programs after the acute phase has beneficial cardiovascular effects such as decrease in arterial stiffness, increased bioavailability of nitric oxide and endothelial function improvement through vasodilatation. Aerobic endurance training, interval training, high intensity interval training and resistance training are the types of exercise training most commonly used [44,45,46].

Studies have shown that in post-COVID-19 patients with moderate illness interval training is preferable and better tolerated as a first approach at an intensity of 2–3 METs with a frequency of 3 to 5 times a week [45,47,48]. High intensity interval training based on series of high intensity exercises interspersed with recovery periods lead to the improvement of endothelial dysfunction through the intensity variation within the same exercise. High intensity exercise programs such as these require a thorough initial evaluation and can be recommended in patients with a high level of fatigue and respiratory distress 2–3 times a week [34,49,50].

Resistance training involves different muscle groups, and it is based on anaerobic exercises of moderate intensity in which resistance is offered by an external load or body weight. Both high intensity interval training and resistance training programs should be adjusted according to the clinical particularities and vitals of the patients [34]. Circuit training programs consist of anaerobic exercises in which all of the muscle groups are included which secondly leads to a more hypotensive response. Compared to endurance training, resistance training is associated with diminished endothelial function improvements and vasodilatation [51,52].

In addition to the benefits mentioned above, there are a number of risks and adverse events during cardiac rehabilitation in post-COVID patients which require further evaluation in order to evaluate the need to participate exercise training programs such as body temperature, heart rate and blood pressure fluctuations or worsening of respiratory symptoms during exercise with no improvement after its discontinuation (Table 2) [36,53].

Respiratory rehabilitation is as important as cardiovascular rehabilitation in patients with CVD and COVID-19. The majority of patients enrolled in exercise training programs associate respiratory problems [36]. Improving ventilation of the deep lung by chest expansion exercises [54,55] and airway clearance by methods based on exploit positive expiratory pressure are the main goals of respiratory rehabilitation which ameliorate dyspnea, reduce morbidity and improves quality of life in patients with COVID-19 and CVD such as PAD [56].

Recovery after COVID-19 can be long, especially for patients with associated comorbidities such as PAD. There are so far unknown long-term effects that influence the quality of life and functional status. These patients may present several limitations in terms of pulmonary dysfunction that do not allow them to undertake a physical effort in the same way as a patient who was never infected.

In the implementation of telerehabilitation, monitoring devices play an essential role through real-time monitoring [57]. Smartwatches, smartphones, pedometers, or sensor devices allow assessment of multiple parameters, both clinical and physical. The impact on quality of life can also be estimated via depression scales, sleep quality monitoring or cognitive tests [5]. Rosen et al. consider telemedicine as an electronic personal protective equipment by reducing both the risks of exposure and contamination between rehabilitation therapists and patients [58]. Group sessions, easy to apply exercise programs and facilitating the access to telerehabilitation for vulnerable patients are practical tips which are easy to apply. Lending devices such as smartphones or smart tables to patients in order to participate in individual session increases adherence among PAD patients enrolled in CR programs [5,59]. As an alternative to traditional exercise programs, during the COVID-19 pandemic patients were encouraged to perform gymnastic movements of muscular strengthening, stretching training and online relaxation sessions previously explained via videos [7,59]. The devices used are often equipped with a series of sensors designed to monitor vital parameters in order to prevent complications and store them in servers easily accessible by healthcare professionals [13,60].

## 4. Physical Training—Mechanisms & Benefits

Loss of exercise tolerance leads to weight gain and influences the lipid metabolism and serum glucose levels, all of which may contribute to the development of atherosclerosis and to an increase in the degree of disability [3].

Increasing exercise capacity is the main goal of physical training in cardiac rehabilitation. The increase in the maximum volume of oxygen (VO2 max) is variable (10–30% usually) and depends on the degree of physical deconditioning of the patient and the intensity of training. Effort adaptation is achieved through central mechanisms such as increases in cardiac contractility or heart rate as well as through peripheral mechanisms such as vasodilation of skeletal muscle arterioles during exercise, an increased oxygen extraction fraction or arterial and venous constriction in the rest of the territories [2,3]. The more trained the patient is, the later the central mechanisms will be involved during the physical training. This reduced vascularization causes a lack in oxygen supply leading to reduced aerobic generation of adenosine triphosphate (ATP) and thus a dependency on anaerobic metabolism. This increases creatine phosphate and lactate levels, which subsequently leads to muscular pain.

**Figure 2:**
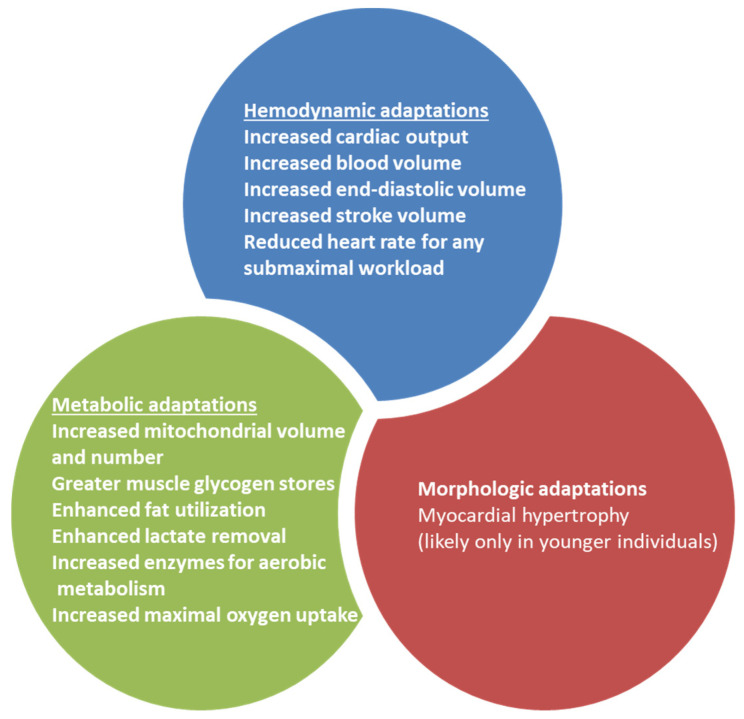
Physiologic adaptations to physical training in humans. Physical training involves hemodynamic, metabolic, and morphologic adaptations.

Physical training induces morphological, hemodynamic, and metabolic changes. The hemodynamic effects are the most obvious after training. The main hemodynamic changes are reduced blood pressure, increased blood volume or increases in maximal oxygen uptake (Figure 2) [61].

Exercise therapy leads to an increase in blood flow through various mechanisms which include but are not limited to arterial collateralization, increased vascular endothelial growth factor levels, increased release of nitric oxide, increased mitochondrial function leading to enhanced oxygen extraction ratios, or decreased endothelial inflammation [62,63].

## 5. Initial Evaluation

Before entering a home-based exercise program, PAD patients willing to be enrolled in a CR program should perform a baseline treadmill cardiac stress test at a specialized center to identify silent or residual ischemia which may lead to an acute event during CR programs. If the stress test indicates coronary ischemia, patients should be further evaluated before being enrolled in a CR program [61]. For PAD patients, a walking assessment is used to establish claudication thresholds and walking time which serve as parameters for determining exercise intensity and subsequently as prognostic indicators.

## 6. Cardiac Rehabilitation—Where Do We Stand

The main objectives of medical and endovascular treatment in PAD patients are to prevent any acute cardiovascular events as well as to improve quality of life appreciated indirectly through physical capacity, stress levels, depression, and social functioning [62].

The addressability or accessibility of PAD patients to CR programs is low and there are several possible explanations for this including the physician, the patient, and health care system levels. Current studies have repeatedly shown that for PAD patients, going to an exercise center for at least 3 times a week for supervised exercise is fairly difficult [3,4,62].

The beneficial role of CR (both functional and social) has been demonstrated in trials based on various exercise programs. Supervised exercise therapy improves cardiorespiratory fitness as well as over-ground and treadmill walking performance in PAD patients, along with a decrease in the mortality rate [63]. An increased exercise capacity is associated with a higher degree of cardiorespiratory fitness and therefore with a superior benefit on morbidity and mortality for PAD patients. CR programs with a low impact on symptomatology should be considered in patients with severe IC or walking disability who cannot participate in supervised programs or have no benefit from it. Women, patients with osteoarticular disease or end-stage renal disease have a higher risk of sedentary behavior [63,64,65,66,67,68,69].

Another issue is that some patients with PAD are reluctant about the efficacy of exercise, especially when it is uncomfortable or painful. The main inconvenience for patients with PAD is pain, followed by low walking capacity with few exercise facilities, and rest places. Alternative modes of exercise combined with low pain exercises increase adherence and participation in CR programs. Claudication increases during walking such that PAD patients need to rest during exercise [62,63,70,71,72,73,74].

Patients with intermittent claudication (IC) usually walk at a moderate speed up to the point of submaximal pain, resuming exercise after the pain has passed. With a total session duration of 30 min (more than 3 times per week), the main objective should be to determine the appearance of IC within 5 min and severe pain within 10 min following rests and repeats. Significant functional improvements are not achieved by walking to near-maximal pain as claudication pain leads to catabolic status with negative impact on skeletal muscles [75,76]. Exercise therapy plays an essential role in the therapeutic management of all patients with PAD except of those with acute arterial occlusion or critical limb ischemia with infection [77].

## 7. Cardiac Rehabilitation Programs

### 7.1. Treadmill Exercise

CR programs based on either supervised treadmill exercise or home-based walking exercise improve walking ability in PAD patients. A total of 3 randomized trials in which a number of 493 patients diagnosed with PAD were included, demonstrated that home-based walking exercise programs combined with behavioral change techniques improves the 6-min walk test performance more than supervised treadmill exercise interventions (45–54 m vs. 33–35 m) as well as walking ability [62,76].

In 1995, Gardner et al. performed a meta-analysis of 21 studies on PAD patients and concluded that supervised treadmill walking improved maximum treadmill walking distance from 125.9 ± 57.3 m to 351.2 ± 188.7 m (*p* < 0.001, increase by 179%) and pain-free treadmill walking distance from 325.8 ± 148.1 m to 723.3 ± 591.5 m (*p* < 0.001, increase by 122%) [78]. They also identified that increases in the distances to onset and to maximal claudication pain during treadmill exercise are independently related to three essential parts of a CR program which can be considered predictors of the changes in claudication pain distances: claudication pain end point used during the exercise training program, the length of the program and the type of exercise. Based on the results from the meta-regression analysis, Gardner et al. concluded that the most effective exercise programs for patients with PAD include 3 sessions per week, 30 min each, at intensity close to the point of maximum or near-maximum pain onset during exercise for at least 6 months [76,79].

Later, in 2012, Fakhry et al. summarized in a meta-analysis the results of 25 randomized clinical trials of supervised walking CR programs in which 1054 symptomatic PAD patients were included. Improvements in both maximal walking distance and pain-free walking distance were achieved in supervised walking exercise group (increase of 180 m, 95% CI, 130–230 m and 128 m, 95% CI, 92–165 m), compared to the control group without exercise. A total of 60% of the trials had a total duration between 12 and 26 weeks. In a subgroup analysis based on the length of the programs (<12 weeks, 12–26 weeks and >26 weeks), using multivariate meta-regression, Fakhry et al. observed the tendency to greater mean improvement in maximum walking distance and pain free walking distance in programs with a duration of 12–26 weeks, that those shorter or longer duration, suggesting a maximum benefit for PAD patients enrolled in CR programs with a duration between 12 and 26 weeks, with 3 sessions per week and 30 min of walking in each session [78,80]. The meta-analysis demonstrated significant functional benefits in treadmill walking performance in patients with PAD after finalizing the supervised CR program, with the reported results suggesting a lower final effect than the one obtained in the meta-analysis reported by Gardner et al. since it included only randomized trials [75,76].

#### 7.1.1. Intensity

It is unclear if walking up to the maximal ischemic pain or rather just up to pain’s onset is more beneficial for the PAD patients, moreover since available trials did not show any difference between these strategies [70].

#### 7.1.2. Program Length

**Figure 3:**
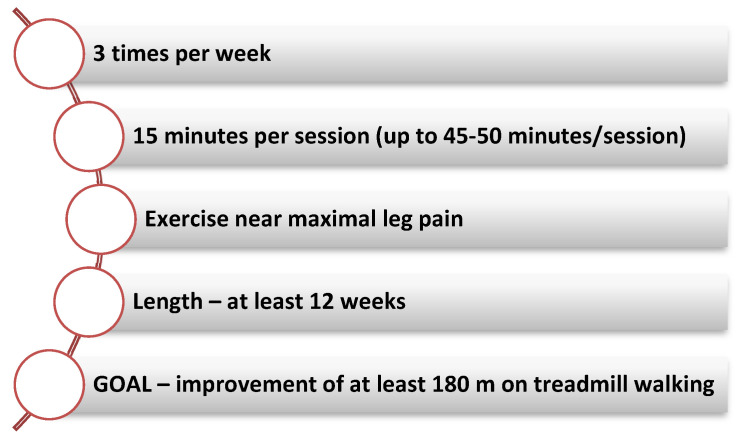
Key elements of an exercise training program.

Fakhry et al. reported significant increases in both pain-free treadmill walking time and maximum treadmill walking time regardless of CR program length (short: 4–11 weeks, medium: 12–26 weeks and long: more than 26 weeks). After 4 weeks of exercise the initial benefit is observed while the maximum benefit of the treadmill walking is achieved after 8–12 weeks of CR. The parameters associated with the 6-min walk test gradually improve due to the fact that the treadmill exercise trains the patient to measure the treadmill walking result (Figure 3) [76].

### 7.2. Home-Based Walking Exercise

Home-based exercise, including behavioral changes, represents an acceptable and affordable alternative to supervised weekly exercise, as it saves time and effort associated to traveling to a dedicated medical center. Home-based walking exercises are more accessible and easier to accept for PAD patients. Regardless of the presence or absence of symptoms, they improve both treadmill walking performance and walk distance in the 6MWT [76,79,81,82]. Furthermore, the benefits of home-based walking sessions in improving both walking capacity and the 6MWT parameters, compared to supervised treadmill exercise programs, have been proved since 2011 through several randomized trials.

Gardner et al. enrolled 119 men and women with symptomatic PAD to 1 of 3 groups (supervised treadmill exercise, home-based walking exercise, or a control group) for a total duration of 12 weeks. Patients randomly assigned to the home exercise group were instructed to perform exercise or walking sessions of at least 45 min, 3 times a week, at their own pace. At the 12-week follow-up, both patients from the home exercise group and the supervised exercise group showed a remarkable improvement in walking distance without the occurrence of IC and an increasing of the maximum exercise duration compared to the control group. Adherence to exercise programs was similar in the 2 groups (*p* > 0.05). PAD patients from the first group walked longer in each session (*p* < 0.001), but with a slower cadence than those in the second group (*p* < 0.05), resulting in a similar total exercise volume, expressed as MET-minutes (*p* > 0.05). No statistically significant differences were identified between the two groups in terms of treadmill walking ability or perimeter walking without IC. It is noteworthy that the study had an overall dropout rate of 23% in the home exercise group and 28% in the supervised treadmill exercise group, pointing difficulties in terms of adherence for PAD patients especially during COVID-19 pandemic [76,79,81,82,83,84].

In the second randomized trial, Gardner et al. randomized 180 PAD patients with IC to 3 groups: supervised treadmill exercise, home-based walking exercise and a light resistance training group. At the 12-week follow-up, patients from the first group had significantly greater improvement in treadmill walking compared to home-based exercise (192 ± 190 s vs. 110 ± 193 s vs. 22 ± 159 s) and in the time to onset of claudication pain on the treadmill (+170 s vs. +104 s vs. +17 s). Beneficial effects were also observed in the 6-minute walking distance which improved by 45 m in the home-based walking group compared to 15 m in the supervised treadmill group and 4 m in the control one [80,83].

The Group Oriented Arterial Leg Study (GOALS) is the only randomized clinical trial of home-based exercise for PAD patients both with and without IC. A total of 192 participants were randomized to a Group Mediated Cognitive Behavioral (GMCB) intervention group or to a control group. The GMCB intervention methods included social cognitive behavioral change theory and group support in order to increase adherence to home-based walking exercise programs and therefore increasing the walking performance. The intervention group had weekly meetings at the medical center with other PAD patients and a facilitator. At the 6 months follow-up, the intervention group had a significantly improved 6-minute walk performance compared to the control group (+42.4 m vs. 11.1 m). Improvements were also observed in the case of pain-free treadmill walking time (+1.01 min compared to the control group) and in maximum treadmill walking time. Support sessions were discontinued after the first 6 months, but the benefits on functional status persisted at the 12-month follow-up [82,85,86].

CR programs for PAD patients should be permanently adapted to the associated comorbidities and needs in order to achieve the desired results. The aim is to achieve a total exercise session duration of up to 50 min, with a gradual increase of 5 min each week, starting from a minimum duration of 30 min per session. The PAD patient should walk until close to reaching maximum leg pain. Even so, trials demonstrated that walking until the onset of intermittent claudication is also beneficial. Rest breaks are acceptable for PAD patients, with the recommendation to resume walking exercise as soon as leg pain has subsided (Table 3) [76,79,81,82,83,84].

Collins et al. also investigated the role of behavioral intervention methods on the adherence of PAD patients to home-based exercise programs. A total of 145 patients with PAD and diabetes were enrolled for 6 months and randomized into 2 groups: a behavioral intervention group vs. an attention control group. The patients from the first group had an individualized counseling session at enrollment, followed by a walking session weekly with an instructor and other patients with PAD at an exercise center and 3 days of walking at home (with a total of 50 min of exercise per session). All patients received bi-weekly phone calls, to evaluate progress in the first group. Compared to the previous study, at the six-month follow-up the investigators found no statistically significant differences in treadmill walking parameters between the two groups [34,82,87].

The impact of COVID-19 pandemic on home-based CR programmes has been assessed by Lamberti et al. in a study in which 83 patients with PAD were enrolled within 9-month before the lockdown. The physical activity consisted of twice a day 8 min sessions of slow and intermittent in-home walking. During lockdown, the patients recevived regular telehone questionnaires regarding general health, adherence to exercise program and evolution of symptoms. Only 80% of the PAD patients showed up for the follow-up after lockdown. The pain-free walking distance improved improved directly proportional to the time since enlistment before the lockdown (*p* < 0.001) regardless of gender and comorbidities. Improvements were also observed regarding body weight, blood pressure and ankle-brachial index [88].

### 7.3. Alternative Forms of Exercise–Ergometry, Cycling and Strength Training

#### 7.3.1. Ergometry

Classic CR programs such as home-based walking exercise or supervised treadmill exercise have more entertaining or challenging alternatives such as cycling, strength training, and upper arm ergometry. Upper-limb exercise determine greater heart rate, intra-arterial blood pressure, and pulmonary ventilation than lower-limb exercise for a specific level of submaximal work [89,90,91]. Multiple randomized trials concluded that both upper- and lower-extremity ergometry have significantly functional benefits especially improving walking endurance in PAD [92].

Zwierska et al. randomized 104 PAD patients into 3 major groups: upper-limb aerobic ergometry, lower-limb aerobic ergometry and a non-exercise control group for a total duration of CR program of 6 months. Each patient performed 2 weekly exercise sessions consisting of 10 cycles of 2 min each of arm (or leg) ergometer cycling, followed by 2 min of rest, with a total duration of 20 min of exercise each session. At the 6-month follow-up, maximum walking distance increased in both upper-limb and lower-limb ergometry groups (29% in the first group and 31% the second group). A beneficial effect was also identified in the peak oxygen uptake, suggesting a link between walking endurance and cardiovascular fitness [84].

Another alternative exercise modality for patients with PAD and IC is arm cranking. Tew et al. randomized a total of 57 patients with IC to an arm-crank exercise group and a non-exercise control group. Patients were evaluated at baseline and at the 12 weeks follow-up. The results showed an incremental improvement to maximum exercise tolerance on both an arm-crank ergometer and a treadmill. In the study group, increases in walking distance (from 496 ± 250 to 661 ± 324 m) and VO2 maximal values (from 17.2 ± 2.7 to 18.2 ± 3.4 mL·kg^−1^ body mass·min^−1^) were recorded in the treadmill walking test (*p* < 0.05). After training, an increase in time to reach minimum tissue O2 saturation (from 268 ± 305 s to 410 ± 366 s), as well as an increase in VO2 kinetics (from 44.7 ± 10.4 to 41.3 ± 14.4 s) and an increase in submaximal StO2 were observed during the treadmill walking test (*p* < 0.05). The increase in walking distance without the occurrence of IC as well as in maximum walking perimeter after arm crank exercise in patients with PAD is partly attributed to improved lower-limb oxygen delivery [93,94,95].

Walker et al. enrolled 67 patients with moderate and severe IC and randomized them in an upper-limb training group (26 patients), a lower-limb training group (26 patients) and a control group (15 patients). The patients from the training group had twice a week 40-min exercise sessions (2 min of exercise followed by 2 min of rest), for 6 weeks. At the follow-up, the pain free walking distance increased by 122% in the first group and by 93% in the second one (*p* < 0.001). Improvements have also been observed in the maximum walking distance which increased by 47% in the upper-limb group (*p* < 0.05) and by 50% in the lower-limb group (*p* < 0.001) [92,96].

#### 7.3.2. Cycling

Lauret et al. included 135 patients from 5 studies that compared different training modalities, including supervised walking exercises. From the results, there was no statistically significant difference regarding reaching the maximum walking distance between supervised walking group and alternative training modalities (8.15 metabolic equivalent <METs>, 95% CI −2.63 to 18.94, *p* > 0.05, equivalent to an increase of 173 m, 95% CI −56 m to 401 m), on the treadmill without incline, for an average speed comparable to everyday life walking (3.2 km/h). At the same time, no statistically significant differences were observed for reaching the maximum walking perimeter without the occurrence of intermittent claudication (6.42 METs, 95% CI −1.52 to 14.36, *p* > 0.05, equivalent to an increase of 136 m, 95% CI −32 m to 304 m), with parameters associated with quality-of-life showing important improvements in both groups [95,97].

Sanderson et al. randomized 42 patients with PAD and IC in 3 different groups: a treadmill exercise group, a cycling one and a control group. The first 2 groups trained 3 times a week for 6 weeks. The exercise consisted of 10 rounds of 2 min each of exercise, interspersed with 2-min breaks, for a total of 20 min of exercise in each session. Cycle training improved cycle performance, but not walking performance. Treadmill training improved maximal and pain-free exercise time by 25%, but not maximal cycle time. While IC in the calf was the most common symptom in the first group, patients from the second group noted the presence of IC in the quadriceps. The difference between the location of IC in the two modes of exercise raise the observation that cycling would not have functional benefit for PAD patients who frequently experience IC in the calf. Sanderson et al. observed in PAD patients with limiting symptoms during cycling and walking a cross-transfer effect between the training modes, suggesting cycling as an exercise alternative for these patients [92,98].

#### 7.3.3. Resistance Exercise Training

Strength training has been used in randomized trials to demonstrate the potential role in improving walking performance for PAD patients. Studies have shown that lower extremity strength training improves maximal treadmill walking time compared to a non-exercise control group. Following resistance training there is an increase in lower extremity skeletal muscle capillary growth. McDermott et al. reported no change in the primary outcome of 6-min walk distance in the strength training group, while supervised treadmill exercise significantly improved 6-min walk distance, leading to the conclusion that walking exercise is more effective than strength training in PAD patients [76,92].

Gomes et al. conducted a randomized controlled trial to evaluate resistance training effects on cardiovascular function. In total, 30 patients with PAD were enrolled and randomly allocated to a control group (15 patients, stretching and relaxation exercises) or resistance training group (15 patients, 3 sets of 10 repetitions of eight whole body exercises, with a pause of 2 minutes between sets). Resting and 24-h blood pressure (BP), cardiac output, systemic vascular resistance, and autonomic variables were obtained before and after 12 weeks of intervention. There was a time effect reduction in heart rate as well as statistically significant changes in cardiac autonomic modulation (*p* < 0.05). In the resistance training group, the blood pressure variability decreased in systolic, diastolic, and mean values (*p* < 0.05). At the 12-week follow-up the resting and 24-h BP, or their hemodynamic and autonomic determinants did not change in the PAD patients enrolled in the training group. However, there were decreases in BP variability, indicating that it could be considered as an alternative to reduce cardiovascular risk (Table 4) [64,76,92,99,100,101].

## 8. Revascularization and Supervised Exercise

Supervised exercise therapy has a positive impact on both functional capacity and quality of life in patients with IC. The combined effect seems to be superior, regardless of order (in a parallel or sequential manner). Studies have shown that supervised exercise has an additional functional benefit in PAD patients. In the randomized ERASE trial, patients were divided into 2 groups: those who received endovascular therapy and additional participated in supervised exercise programs (106 patients) and those who were enrolled in CR programs based on supervised exercise alone (106 patients). At the one-year follow-up, in both groups functional and quality-of-life related parameters were achieved, both groups showing improvements in maximal walking distance, pain-free walking distance, and quality of life.

Fakhry et al. demonstrated in a randomized clinical trial of 212 patients (allocated to either endovascular revascularization and supervised exercise or supervised exercise only) that among patients with IC, at the 1-year follow-up, endovascular revascularization followed by supervised exercise had better results in walking distances and quality-of-life scores than supervised exercise only [76,83,102]. Combined therapy was associated with a significantly greater improvement in maximum walking distance and pain-free walking distance. Regarding the maximum walking distance, the mean difference between groups was of 282 m (improvement of 1237 m vs. 955 m) (99% CI, 60–505 m). The mean difference was bigger for the pain-free walking distance (408 m) with an improvement of 1120 m, respectively 712 m for the combined therapy group or supervised exercise only (99% CI, 195–622 m). A significantly greater improvement was also obtained in the disease-specific VascuQol score in both groups (1.34 in the combination therapy group vs. 0.73 in the exercise group; mean difference, 0.62 [99% CI, 0.20–1.03]) as well as in the score for the SF-36 physical functioning questionnaire (22.4 vs. 12.6 respectively; mean difference, 9.8 [99% CI, 1.4–18.2]). There were no differences in the SF-36 domains of physical role functioning, bodily pain and general health perceptions between the 2 groups [65,102].

## 9. Effects on Biomarkers after Cardiac Rehabilitation

Home-based exercise programs which can be overseen have multiple vascular benefits in patients with PAD with IC by ameliorating various biological markers such as blood glucose, circulating markers of endogenous antioxidant capacity, as well as inflammatory related to the inflammation of the endothelium. E-selectin, intercellular adhesion molecule (ICAM-1), and interleukin-6 (IL-6) are some of the vascular and inflammatory biomarkers reduced after participating in supervised CR programs [102]. Previous studies suggested that this effect is inconsistent on high-sensitivity C-reactive protein (hsCRP) [103,104]. Gardner et al. randomized 114 patients into 3 groups different by the intensity of exercise programs and supervision level: home-based programs, supervised programs of low intensity resistance training and walking to mild-to-moderate claudication pain for 12 weeks as well as a control group with unsupervised training. Upon enrolment and after finishing the 12-week exercise program, treadmill performance was evaluated, as well as circulating inflammatory biomarkers known the endothelial effects of circulating factors, using a cell culture-based bioassay on primary human arterial endothelial cells [83,104]. The statistical analysis showed that treadmill peak walking time improved in all groups except the control group (*p* < 0.05). Cultured endothelial cell apoptosis diminished in the first group (exercises performed at home, *p* < 0.05). The antioxidant capacity of the hydroxyl radical (HORAC) (*p* < 0.05) increased as well as the vascular endothelial growth factor A levels (VEGF-A) (*p* < 0.05). In particular, it was observed in patients from the first group that E-selectin (*p* < 0.05) and blood glucose levels decreased (decreases insulin resistance) (*p* < 0.05) [105,106,107,108].

In patients with IC or other PAD symptoms, the daily activity level was positively correlated with HORAC, suggesting that ischemic preconditioning is ameliorated after daily exercises due to circulating antioxidant capacity. At the eight weeks follow-up E-selectin levels decreased after supervised exercise training, but studies suggest that this result is inconsistent. Gardner et al. demonstrated that VEGF-A serum levels increased after home-based exercise. A non-randomized exercise trial found that VEGF-A serum levels were not modified after following non-supervised exercise programs but increased initially with a program of supervised exercise. Previous studies demonstrated that skeletal muscle VEGF-A was decreased following 12 weeks of supervised exercise and remained unchanged after a home-based exercise program [83].

Blood glucose level decreases after a home-based exercise program and does not change following supervised treadmill exercise. Its clinical significance is based on elevated fasting glucose in patients with symptomatic PAD being associated with peripheral circulation, patient-perceived walking ability, health-related quality of life, and sedentary behavior. Diabetes impairs microcirculation and metabolic syndrome impairs ABI and claudication distances in patients with PAD [106,107,108,109,110,111].

## 10. Physical Exercise Protocols

Exercise is an essential part of CR programs for PAD patients, with most functional benefits being observed in the first 2–3 months of supervised training. Current studies demonstrated that PAD patients should exercise at least 3 times every week, for about 30–60 min, with a total training volume of 1500–2000 min. Wang et al. demonstrated that exercise using an individual leg plantar flexion ergometer improves peak oxygen consumption (VO2), at that level 78% of the patients enrolled being symptom free [111,112].

Lower-limb resistance training based on knee extension and leg press/curl exercises is an alternative to treadmill workout, studies suggesting that 1 repetition maximum improves the 6-minute walk test distance by 12.4 m comparing to a control group. PAD patients who associate important comorbidities such as heart failure and have difficulties in walking on a treadmill can use lower-limb resistance training as an alternative to treadmill exercises with a diminished total effect on symptoms, especially IC [111,112,113].

Upper-limb cycle ergometry ameliorates the walking distance and can be used as an alternative training method in PAD patients with functional impairment due to symptoms associated with IC. Its positive effect on symptoms suggests the presence of a cross-training effect on the lower-limb through several adaptive mechanisms. Further studies are much needed to determine the physiological mechanism [114,115,116].

Pole striding involves both upper- and lower-limb exercises. Its functional benefit was illustrated in a randomized controlled trial by improving both peak VO2 and pain-free walking distance in comparison to placebo or vitamin E groups. Taking into consideration the fact that it involves both upper- and lower-limb muscles, this alternative method of training may have additional benefits which require further research. Which types of exercises have the maximum functional effect, in which combination or duration, are some of the main questions needed to be answered in the near future in order to determine the best strategy regarding CR programs with maximum benefit in reducing IC symptoms and improving the long-term prognosis [116,117,118,119,120,121].

## 11. Psychological and Social Outcome

CR programs have multiple functional benefits and a great impact on the psychosocial status, increasing quality of life, decreasing depression and anxiety as well as reducing the stress levels [26]. After being part of a CR program, PAD patients testified that their energy levels increased. Unfortunately, up until now, studies regarding PAD patients enrolled in different types of CR programs have offered little information about psychosocial outcomes and although exercise programs have positive impact on the quality of life, further research on this topic are needed [63,92].

Stauber et al. coordinated a study in which 529 patients were included in a comprehensive 12-week outpatient CR program. All of the patients fulfilled psychosocial questionnaires upon inclusion in the study and at the 12-week follow-up. Hospital Anxiety and Depression Scale questionnaire (HADS) was used for analyzing the depression and anxiety levels of PAD patients, while the positive and negative impact of the cardiovascular disease was monitored with the Global Mood Scale. Different health-related aspects with impact on the quality of life were measured with the SF-36 Health Survey. The 12-week follow-up proved that exercise training programs had multiple benefits on anxiety (*p* < 0.001) and negative affect (*p* < 0.001), as well as in bodily pain as a health-related quality of life marker (*p* < 0.001) [3,4,120,121]. The 6MWT distance is independently and statistically associated with better physical and social aspects of psycho-emotional status in people with IC [122]. In terms of mental health and quality of life, COVID-19 has a negative impact. PAD typically limits social activities and interpersonal relations.

De Donato et al. enrolled 102 patients with PAD and, through a questionnaire, evaluated the quality of life between the “No-COVID-19 period” (July–December 2019) and the pandemic time period (January–June 2020). An increase in pain intensity has been highlighted during SARS-CoV2 pandemic (from a mean score of 4.7 ± 2.9 to 6.3 ± 2.9, *p* < 0.0001) but without an increase in the consumption of analgesic drugs (*p* = 0.15). The overall lower-limb health status worsened (*p* = 0.0001) due to the restrictions which limited the possibility of walking outside combined with the fear of infection in public places. All of these aspect influence mental health and have caused among patients enrolled in the study an increasing of the feeling of fear associated with disease worsening (*p* < 0.0001) [123].

## 12. Conclusions

Physical exercise remains an important element of rehabilitation in patients with CVD. The management of PAD during the COVID-19 pandemic has been a major challenge for both patients and medical personnel. Supervised exercise therapy has a positive impact on both functional capacity and quality of life in PAD, although the combination with revascularization therapy seems to be even more effective. Improving functional capacity, increasing quality of life, and preventing depression are some of the benefits seen in patients with PAD included in CR programs.

## Figures and Tables

**Table 1:** Parameters evaluated during the 6 Minute Walking Test after COVID-19 (adapted after [34]).

Parameters	Value
Saturation	>92–93%
Heart rate	An increase up to 20 beats per minute from the baseline
Systolic blood pressure	≥90 mmHg and ≤180 mmHg
Symptoms	The Borg Scale–dyspnea with a score < 4 Rate of perceived exertion–fatigue with a score < 11–12

**Table 2:** Adverse hemodynamic events during cardiac rehabilitation, especially after COVID-19 (adapted after [34]).

Parameters	Value
Saturation	<88–93%
Heart rate	<40 beats per minute or >120 beats per minute
Systolic blood pressure	<90 mmHg and >180 mmHg
Body temperature fluctuations	>37.2 °C
Symptoms	Worsening of respiratory symptoms during exercise Chest tightness or pain Difficulty in breathing Palpitations Sweating
	Unclear vision

**Table 3:** Change in Walking Distance after cardiac rehabilitation programs in patients with PAD. For PAD patients enrolled in different cardiac rehabilitation programs, there is a direct proportionality between duration and claudication onset distance or change in peak walking distance.

Supervised Program (Studies until 2021)	Claudication Onset Distance (Mean ± SD, %)	Peak Walking Distance (Mean ± SD, %)
12 weeks (8 studies)	156.60 ± 46.97 m (103%)	283.10 ± 69.32 m (79%)
24–52 weeks (7 studies)	251.23 ± 75.72 m (167%)	334.06 ± 78.14 m (92%)
Overall (15 studies)	203.93 ± 77.93 m (128%)	307.45 ± 75.58 m (82%)

**Table 4:** Comparison of resistance exercise training programs in patients with PAD. High-intensity resistance training is superior to treadmill exercise training in improving walking distance in PAD patients.

	Group	Increase Peak Walking Distance (Mean)	Increase Claudication Onset Distance (Mean)	Increase the 6 Minute Walking Test Distance (Mean)
Hiatt et al. [99]	Resistance Training Group	+107 m	+1.6 m	NA
	Treadmill Walking Group	+273 m	+182	NA
McDermott et al. [76]	Resistance Training Group	+129 m	+102	−3 m
	Treadmill Walking Group	+212 m	+156 m	+20.9 m
Ritti-Dias et al. [100]	Resistance Training Group	+157 m *	+146	-
	Treadmill Walking Group	+149 m *	+127 m	-
Parmenter et al. [101]	Resistance Training Group	-	-	+60 m
	Treadmill Walking Group	-	-	−9 m

* Change with high-intensity resistance training.
